# Tinnitus and hyperacusis therapy in a UK National Health Service audiology department: Patients’ evaluations of the effectiveness of treatments

**DOI:** 10.1080/14992027.2016.1178400

**Published:** 2016-05-19

**Authors:** Hashir Aazh, Brian C. J. Moore, Karen Lammaing, Mark Cropley

**Affiliations:** ^a^ Audiology Department, Royal Surrey County Hospital NHS Foundation Trust, Guildford, UK; ^b^ Department of Experimental Psychology, University of Cambridge, UK; ^c^ School of Psychology, University of Surrey, Guildford, UK

**Keywords:** Tinnitus, hyperacusis, education, client-centered counselling, hearing aids, sound therapy

## Abstract

*Objective*: To assess patients’ judgements of the effectiveness of the tinnitus and hyperacusis therapies offered in a specialist UK National Health Service audiology department. *Design*: Cross-sectional service evaluation questionnaire survey. Patients were asked to rank the effectiveness of the treatment they received on a scale from 1 to 5 (1 = no effect, 5 = very effective). *Study sample*: The questionnaire was sent to all patients who received treatment between January and March 2014 (n = 200) and 92 questionnaires were returned. *Results* **:** The mean score was greatest for counselling (Mean = 4.7, SD = 0.6), followed by education (Mean = 4.5, SD = 0.8), cognitive behavioural therapy - CBT (Mean = 4.4, SD = 0.7), and hearing tests (Mean = 4.4, SD = 0.9). Only 6% of responders rated counselling as 3 or below. In contrast, bedside sound generators, hearing aids, and wideband noise generators were rated as 3 or below by 25%, 36%, and 47% of participants, respectively. *Conclusion*: The most effective components of the tinnitus and hyperacusis therapy interventions were judged by the patients to be counselling, education, and CBT.

Tinnitus is the sensation of sound in the ears or head without actual acoustic stimulation. Hyperacusis is a term that is used to describe intolerance to everyday sounds that causes significant distress and impairment in social, occupational, recreational, and other day-to-day activities. The sounds may be perceived as uncomfortably loud, unpleasant, frightening, or painful. Several authors and patient groups have given other definitions for hyperacusis. For details see Aazh et al (2014); Jastreboff and Jastreboff (2014); Tyler et al (2014).

Audiology departments in the UK National Health Service (NHS) play a major role in offering therapy and support for patients experiencing tinnitus and hyperacusis. A Medical Research Council (UK) study of ear-related symptoms in the UK population reported that 42% of patients whose mean audiometric threshold across 0.5, 1, 2 and 4 kHz was ≥45 dB HL had prolonged tinnitus (Coles, 1984). Connections between hyperacusis, hearing impairment, and tinnitus have been highlighted by several authors (Formby et al, 2007; de Klaver et al, 2007; Schecklmann et al, 2014; Tyler & Conrad Armes, 1983). Schecklmann et al (2014) reported that 67% (308/921) of patients with hyperacusis combined with tinnitus reported hearing problems, compared to 57% (327/767) of patients with tinnitus only. The relationship between tinnitus, hyperacusis, and hearing impairment probably explains why, in the UK, 82% of tinnitus patients are referred to Audiology departments for treatment, either via their general practitioners (GP) or via Ear-Nose-Throat specialists (Gander et al, 2011). The referral pathway for hyperacusis patients is less understood but it is likely that they too mainly are referred to Audiology departments for treatment.

A good practice guide (Department of Health, 2009) on the provision of services for adults with tinnitus recommends the use of structured interviews, audiological investigations, and psychometric self-report questionnaires in the assessment of tinnitus. For tinnitus management the guide recommends education/information, hearing aids, psychological support, relaxation therapy, cognitive behavioural therapy (CBT), sleep management, and sound therapy. Hoare et al (2012) reported that almost all tinnitus services in the NHS in England (practice may differ in other parts of the UK) offer combinations of the above therapies. Ninety-one percent of services reported that they routinely conduct audiometry as a part of the assessment process, 67% use validated questionnaires, 99% of those conducting audiometry offer hearing aids (for tinnitus combined with hearing loss), 96% offer education/information, 96% offer sound generators, and 46% offer CBT (Hoare et al, 2012). Recently, the American Academy of Otolaryngology - Head and Neck Surgery published a clinical practice guideline for tinnitus (Tunkel et al, 2014). Consistent with the UK good practice guide, they recommend history taking, audiological examinations, education about management strategies, hearing aid evaluation, and CBT. Sound therapy was not recommended, but was suggested as an optional treatment.

To the authors’ knowledge there is no widely agreed guideline for the management of hyperacusis. A conference report suggests that various forms of counselling and sound therapy seem to be beneficial in the management of hyperacusis, but the evidence base for these remains poor (Aazh et al, 2014).

Randomized controlled trials are needed to evaluate the effectiveness of the various forms of intervention that are used to treat tinnitus and hyperacusis (Pocock, 1983). However, complementary to this, it is useful to assess how patients view the effectiveness of the various interventions. For example, if a patient with tinnitus is fitted with hearing aids but finds them to be ineffective in relation to their tinnitus, the patient might stop wearing the hearing aids, which would be a waste of resources. In addition, patients’ views on the effectiveness of interventions may be passed on to other patients who are just starting treatment (for example via self-help groups or social media) and this may influence the new patients' willingness to undergo certain interventions and might bias their expectations. Finally, as reviewed below, the evidence base for many of the interventions is weak, and in the absence of good evidence patients’ views become relatively more important.

This paper presents the results of a survey providing information about patients’ views of the effectiveness of the interventions offered by the Tinnitus & Hyperacusis Therapy Specialist Clinic (THTSC) at the Royal Surrey County Hospital (RSCH), which is an audiologist-led service. Consistent with the guidelines (Tunkel et al, 2014; Department of Health, 2009) and similar to other tinnitus services in England, the THTSC offers a mixture of interventions comprising: (1) education, (2) CBT, (3) hearing aids (4) sound therapy, and (5) client-centred counselling. A brief description of each intervention and its evidence base is given below, although, as noted above, the emphasis of this paper is on patients’ views of effectiveness rather than on the evidence base for the interventions.

## Education

Educational sessions' content at THTSC is informed by tinnitus retraining therapy (TRT; Jastreboff & Hazell, 2004). Patients are offered detailed information about (1) the outcome of their audiological and otological investigations, (2) the basic functions of the auditory system, (3) the basics of brain function and the interactions of the various systems of the brain, and (4) the theoretical basis of habituation based on the Jastreboff neurophysiological model (Jastreboff & Hazell, 1993). All audiologists use a PowerPoint slide presentation to provide education. The presentation was developed based on the educational component of TRT and covers tinnitus as well as hyperacusis (depending on the patient’s need). It should be noted that some aspects of this type of education were used in the treatment of tinnitus and hyperacusis prior to the development of TRT.

There are two systematic reviews supporting the efficacy of educational sessions based on the TRT approach in combination with sound therapy in the management of tinnitus (Grewal et al, 2014; Phillips & McFerran, 2010). However, the evidence base for the efficacy of education alone in management of tinnitus or hyperacusis is poor.

## Cognitive behavioural therapy (CBT)

Cognitive behavioural therapy (CBT) is a psychological intervention that aims to help the patient to modify their unhelpful, erroneous cognitions and safety-seeking behaviours (Beck, 1976; Clark et al, 1999). The CBT approach is collaborative, with a strong emphasis on the clinician and patient working on a problem together. One goal is to test reality through ‘collaborative empiricism’, whereby the clinician and patient work together to test a range of hypotheses. Throughout, the principle of guided discovery is employed, in that the patient makes discoveries with some guidance from the clinician rather than the clinician pointing out maladaptive behaviour or errors in thinking. CBT involves helping the patient to identify, challenge, and modify their unhelpful thoughts in response to tinnitus or environmental sounds. Use of CBT in the management of tinnitus and hyperacusis has been recommended by several authors (Pienkowski et al, 2014; Tyler et al, 1989). The CBT techniques at THTSC comprised: Socratic questioning, guided discovery, behavioural experiments, education, and filling in diaries of thoughts and feelings between the sessions.

There is a wide range of research supporting the efficacy of CBT in the management of tinnitus (Hesser et al, 2011; Grewal et al, 2014). The authors are aware of only one published research study on hyperacusis management that reports some benefits from CBT (Juris et al, 2014).

## Hearing aids

Hearing aids are offered to patients if they have tinnitus combined with self-reported hearing difficulties and a hearing loss that could be helped with hearing aids. There is no specific guideline on the minimum hearing loss that warrants fitting of hearing aids. The decision is typically made based on the audiologist’s clinical judgment. The hearing aids used were Danalogic i-fit 71TS behind-the-ear combination devices with a volume control. Hearing aids were fitted to NAL-NL2 (Keidser et al, 2012) targets verified by real-ear measurements, conducted following the British Society of Audiology guidelines (BSA, 2007). Three programs were entered into each device comprising: (1) amplification only, (2) amplification plus wideband noise, and (3) wideband noise only. Patients could select programs as desired. The instructions were to use the device as much as possible during waking hours. The level of the wideband noise was set just below the ‘mixing point' as described by the TRT protocol (Jastreboff & Hazell, 2004). No attempt was made to adjust the spectrum of the noise based on the characteristics of the tinnitus.

Despite their widespread use, there seem to be conflicting results with regard to the effectiveness of hearing aids in the management of tinnitus. A recent Delphi review suggested that there is disagreement among clinicians with regard to the fitting of hearing aids for patients with tinnitus combined with a mild hearing loss (Sereda et al, 2015). While several authors have recommended the use of hearing aids in tinnitus management (Henry et al, 2015; Moffat et al, 2009), a recent Cochrane systematic review concluded that there is currently no evidence to support or refute their use as a routine intervention for tinnitus (Hoare et al, 2014). Moreover, another review did not find evidence supporting or refuting the efficacy of hearing aids in reducing tinnitus handicap for people with hearing loss and tinnitus, except for one low-quality randomized controlled trial (Melin et al, 1987), which suggested that hearing aids may be no more effective at reducing the severity of tinnitus after six weeks than being on a waiting list (Savage & Waddell, 2014).

Consistent with the TRT protocol, patients with hyperacusis combined with hearing loss were instructed to start with the wideband-noise-only program and to move on to the hearing aid program after they had shown improvement in management of their hyperacusis (Jastreboff & Hazell, 2004). The procedure for fitting and verification of hearing aids was similar to that for tinnitus, as described above. There is very limited research about the effectiveness of fitting of hearing aids for patients with hyperacusis combined with hearing loss (Pienkowski et al, 2014).

## Sound therapy

For sound therapy, all patients were offered bedside sound generators (SGs) to use at night. These provided a range of sounds, and the patient could select the sound that they thought worked best for them. For use during the day, patients with no hearing loss were offered the Danalogic i-fit 71TS combination devices using thin tubes (open-fit). These acted as wearable noise generators without any amplification (recall that those with hearing loss were offered the same devices but with three programs, including two with wideband noise). The setting with no amplification is called here a wideband noise generator (WNG). Bilateral devices were offered to all patients. The instructions for sound therapy followed the TRT protocol, as described above (Jastreboff & Hazell, 2004).

Research supporting the effectiveness of sound therapy for tinnitus or hyperacusis is limited, as in most studies sound therapy has been offered in combination with educational sessions (Hobson et al, 2012; McKenna & Irwin, 2008; Pienkowski et al, 2014).

## Client-centred counselling

Client-centred counselling was developed by Carl Rogers (Rogers, 1951) and emphasizes respecting and trusting the patient’s capacity for growth, development, and creativity (Rogers, 1959). Empathic listening is a key counselling skill that is used throughout the therapy sessions to build a good patient-clinician relationship and offer emotional support to patients. Empathy means to understand and feel another person’s perspectives (Rogers, 1959). This is different from sympathy and is completely opposite to imposing one’s own views with the assumption that the patient’s views are inaccurate or misguided (Rollnick & Miller, 1995). Empathic listening involves asking open-ended questions, focusing, paraphrasing, reflecting on meanings, reflecting on feelings, structuring, and summarizing (Jenkins, 2000). The idea is that listening empathically to a patient’s story and concerns will promote their capacity for self-growth and acceptance of tinnitus or their ability to tolerate sound. Use of client-centred counselling in the management of tinnitus and hyperacusis has been recommended by several authors (Pienkowski et al, 2014; Tyler et al, 2001). However, to the author’s knowledge there is no study in the literature that assesses the effectiveness of client-centred counselling in the management of tinnitus or hyperacusis.

To sum up, evidence for the effectiveness of hearing aids and sound therapy, which are offered routinely by tinnitus services, including THTSC, is limited. Moreover, the application of client-centred counselling, which is used in all therapy sessions at THTSC, does not seem to have any evidence base. The aim of the present service evaluation study was not to evaluate the efficacy of the various aspects of the treatment. Rather, the aim was to obtain feedback from patients about their views of the effectiveness of the treatment they received for tinnitus and hyperacusis at THTSC. The information was intended to complement research findings and guide the provision of care for patients with tinnitus and hyperacusis.

## Method

### Study design

This was a cross-sectional service evaluation survey. The RSCH has a catchment area of 320 000 people. According to local service agreements, all patients who need tinnitus or hyperacusis therapy should be referred via their GPs or other health professionals. The THTSC receives approximately 40 new referrals per month. Between January 2014 and January 2015, 739 patients were seen (combination of new and existing patients). The survey was conducted between May and June, 2015. In order to include patients who had received therapy for at least 12 months, the survey questionnaire was sent to all patients who were initially seen between January and March, 2014.

The study was approved by the Audiology Department at the RSCH and was registered with the Clinical Audit, Patient Safety & Quality department.

### Study population

The average age of the patients (*n* = 200) was 57 years (standard deviation, SD =18 years, range 7 to 95 years). Fifty-four percent of patients were male (*n* = 108). At the time of the survey, on average patients were seen for six sessions (SD =4.5, range 1 to 23). The mean pure-tone average (PTA) audiometric threshold across the frequencies 0.25, 0.5, 1, 2, and 4 kHz was 25 dB HL (SD =17 dB) for the right ears, and 27.5 dB (SD =19 dB) for the left ears. Means and SDs of scores on the tinnitus handicap inventory (THI; Newman et al, 1996), the hyperacusis questionnaire (HQ; Khalfa et al, 2002), the hospital anxiety and depression scale (HADS; Zigmond & Snaith, 1983), the visual analogue scale (VAS; Maxwell, 1978) of tinnitus loudness, annoyance, and effect on life, and the insomnia severity index (ISI; Morin, 1993) as measured in the initial pre-treatment assessment session, are shown in Table 1.

**Table 1.** Means and SDs of the pre-treatment scores on the tinnitus handicap inventory (THI), hyperacusis questionnaire (HQ), hospital anxiety and depression scale (HADS), visual analogue scale (VAS), and insomnia severity index (ISI) questionnaires for patients seen between January 2014 and March 2014 at THTSC.

Variable	N	Mean	SD
THI	169	45	23
VAS (Tinnitus loudness)	163	6	2
VAS (Tinnitus annoyance)	164	6	2
VAS (Effect on life)	164	5	3
HADS (Anxiety)	172	9	5
HADS (Depression)	172	6	4.5
ISI	152	12	7
HQ	164	18	9

### Setting and current practice

At THTSC, all patients undergo an assessment which comprises:

1. Taking a case history
2. Ear examination using an otoscope
3. Pure-tone audiometry based on the procedure described by the British Society of Audiology (BSA, 2004)
4. Measurement of uncomfortable loudness levels (ULLs) following the BSA recommended procedure (BSA, 2011)
5. A wide range of self-report questionnaires including the THI, HQ, ISI, HADS, and the VAS. These are described in more detail below.

Patients who do not meet the British Academy of Audiology criteria (BAA, 2007) for direct referral from GPs are referred to ENT or audiological medicine for further otological examination. Patients are offered individual face-to-face therapy sessions with audiologists who are specialized in tinnitus and hyperacusis rehabilitation. Each therapy session lasts between 60 and 90 minutes. Therapy comprises a mixture of (1) education, (2) CBT, (3) hearing aids, (4) sound therapy, and (5) client-centred counselling.

### Training of the audiologists

The three audiologists who delivered the therapy had been trained in tinnitus and hyperacusis rehabilitation via attending a five-day tinnitus and hyperacusis therapy masterclass. This was a practical training course focusing on (1) client-centred counselling skills, (2) basic CBT skills, (3) education based on the TRT protocol, and (4) sound therapy based on the TRT protocol. The course involved 30 hours of direct contact, 100 hours of directed self-study (i.e., reading and working through the provided/ recommended course materials), and 20 hours of self-directed learning (i.e., general reading around the subject, and contributing to online discussion forum). After attending the course, the three audiologists received six months of supervised practice during which they had the opportunity to observe therapy sessions in the clinic and to deliver therapy under direct supervision. After these six months, they received ongoing coaching and clinical supervision from the first author, during which they could discuss their difficult patients and receive feedback and additional informal training when indicated. In addition, the audiology department at the RSCH supports continuous professional development for staff members, assisting them to take part in various conferences and short courses on the topics of tinnitus, hyperacusis, and psychological therapies each year. Audiologists also benefited from regular team meetings where they could share their concerns and ideas with their peers.

### Questionnaires

#### Questionnaire for the service evaluation survey

The questionnaire included nine items assessing patients’ opinions of the effectiveness of the therapies that they received (see Appendix 1). Patients were asked to rate the effectiveness of each therapy on a scale from 1 to 5 (1 = no effect, 5 = very effective). They were instructed to leave the form blank if they had not received a specific therapy. The questionnaire items were concerned with: (1) Hearing tests, (2) Completing the tinnitus/hyperacusis questionnaires, (3) Education and information about tinnitus/hyperacusis, (4) Counselling, (5) CBT, (6) Bedside SG, (7) WNG, (8) Hearing aids, and (9) Overall satisfaction with the tinnitus/hyperacusis clinic. In addition, patients were asked whether they had tinnitus (yes/no), hearing loss (yes/no), or hyperacusis (yes/no), and to specify the duration of their tinnitus. Patients were asked to return the questionnaire within two weeks, using the pre-paid envelope provided. No reminders were sent.

#### Other questionnaires used routinely in the clinic

The THI has 25 items, and response choices are ‘no' (0 points), ‘sometimes' (2 points), and ‘yes' (4 points). The overall score ranges from 0 to 100. Scores from 0–16 show no handicap, scores from 18–36 show mild handicap, scores from 38–56 indicate moderate handicap, and scores from 58–100 show severe handicap (Newman et al, 1998).

The HADS consists of 14 items each rated from 0 to 3 according to severity of difficulty experienced. Eight items require reversed scoring, after which depression (HADS-D) and anxiety (HADS-A) subscale totals can be obtained. Total scores for each subscale range from 0 to 21. Scores from 0–7 are classified as normal, scores from 8–10 are classified as borderline abnormal, and scores from 11–21 indicate abnormal (Zigmond & Snaith, 1983).

The ISI comprises seven items that assess the severity of sleep difficulties and their effect on the patient’s life. Each item is rated on a scale of 0 to 4 and the total score ranges from 0 to 28. Scores from 0–7 indicate no clinically significant insomnia, scores from 8–14 indicate minimal insomnia, scores from 15–21 indicate moderate insomnia, and scores from 22–28 indicate severe insomnia (Bastien et al, 2001).

The HQ comprises 14 items and the response choices are ‘no' (0 points), ‘yes, a little' (1 point), ‘yes, quite a lot' (2 points), and ‘yes, a lot' (3 points). The overall score ranges from 0 to 42. Scores above 28 indicates strong auditory hypersensitivity (Khalfa et al, 2002).

VAS scores are ratings on a scale from 0 to 10. The VAS score for loudness of tinnitus was assessed by asking the patient to rate the loudness of tinnitus during their waking hours over the last month (It was explained that 0 corresponds to no tinnitus being heard and 10 is as loud as gunfire). The VAS score for annoyance induced by the tinnitus was assessed by asking the patient to rate their subjective perception of annoyance on average during the last month (It was explained that 0 corresponds to no annoyance and 10 is the most annoying thing that can possibly happen). The VAS score for the impact of tinnitus on their life was assessed by asking the patient to rate the effect of tinnitus on their life during the last month (It was explained that 0 corresponds to no effect and 10 is as big as an earthquake).

### Data analysis

Participants’ ages, gender, pure-tone audiograms, ULLs, number of therapy sessions received, and scores on assessment questionnaires were imported from the records held in the electronic database of the RSCH Audiology Department. The data were anonymized prior to statistical analysis. Descriptive statistics were calculated for items on the questionnaires and patients’ characteristics. Group differences between responders and non-responders were assessed using t-tests. The differences between the items of the survey questionnaire were assessed using Wilcoxon signed-rank tests. The differences between subgroups of patients based on the presence/absence of hyperacusis and among the audiologists were assessed using Mann-Whitney U tests and Kruskal-Wallis tests, respectively. In order to account for the number of comparisons being made, the *p* value required for statistical significance was set at *p* < 0.005. The STATA programme (version 13) was used for statistical analyses. The analyses were restricted to responders with complete data on all variables required for a particular analysis.

## Results

### Responders versus non-responders

A total of 92/200 questionnaires were returned, a response rate of 46%. The mean number of therapy sessions received at the time of the survey was 8 (SD = 5) for the responders and 5 (SD = 4) for non-responders (*p* < 0.001) (Table 2). Sixty-three percent (58/92) of responders and 46% (50/108) of non-responders were male (*p* = 0.02). As shown in Table 2, non-responders were younger than responders (*p* < 0.001) and their mean PTA for the better ear was better (*p* = 0.02). The mean ULL, averaged across ears and over the frequencies 0.25, 0.5, 1, 2, 4, and 8 kHz was 88 dB HL (SD = 12) for the responders and 84 dB HL (SD = 14) for non-responders (*p* = 0.15). There were no significant differences between the responders and non-responders in scores for the THI, VAS, HQ, HADS, and ISI questionnaires (Table 2).

**Table 2.** Comparison of the means and SDs of age, pure-tone average, and scores on the self-report questionnaires obtained in the initial assessment session for the responders and non-responders.

	Responders (n)	Responders mean (SD)	Non-responders (n)	Non-responders mean (SD)	*p*-value
Age, years	92	62 (15)	108	52 (19)	<0.001
Number of therapy sessions attended	92	8 (5)	108	5 (4)	<0.001
PTA of better ear (dB HL)	87	25 (17)	98	20 (14)	0.02
PTA of worse ear (dB HL)	85	32 (22)	97	29 (18)	0.23
Tinnitus handicap inventory (range 0–100)	86	47 (24)	83	44(23)	0.32
Hyperacusis questionnaire (range 0–42)	82	18 (8)	82	18(9)	0.98
Visual analogue scale of tinnitus loudness (range 0–10)	81	6 (2)	82	6 (2)	0.85
Visual analogue scale of tinnitus annoyance (range 0–10)	82	6 (2)	82	6 (2)	0.94
Visual analogue scale of effect of tinnitus on life (range 0–10)	82	6 (2)	82	5 (3)	0.06
Insomnia severity index (0–28)	78	13 (7)	74	12 (7)	0.46
Hospital anxiety and depression scale: Anxiety domain (0–21)	87	9 (4)	85	9 (5)	0.66
Hospital anxiety and depression scale: Depression domain (0–21)	87	6 (5)	85	6 (4)	0.7

### Characteristics of responders

The mean duration of tinnitus for the responders was 10 years (SD =10). Ninety-six percent (89/92) of the responders reported having tinnitus, 39% (36/92) reported having hyperacusis, and 72% (66/92) had hearing loss. Forty-eight percent of the responders (44/92) had received bedside SGs, 64% (59/92) ear level devices (i-fits) incorporating a WNG, and 60% (55/92) had received i-fits incorporating amplification.

### Effect of the treatments from the patients’ perspective

Each item on the questionnaire was rated as 4/5 or 5/5 (very effective) by over 50% of the responders (Table 3). The mean score was greatest for counselling, followed by education, CBT, and hearing tests. Only 6% of responders rated counselling as 3/5 or below. This was followed by education, hearing tests, and CBT, which only 9%, 12%, and 15% of responders rated as 3/5 or below, respectively. This is in contrast with the bedside SGs, hearing aids, and WNGs, which 25%, 36%, and 47% of responders rated as 3/5 or below, respectively.

**Table 3.** Summary of responses to the nine items on the service evaluation survey questionnaire. Mean scores (and SDs) are also given for each item.

Item	Please rank the effect of the treatments you received with regard to the management of your tinnitus or hyperacusis.	Answer	Number (%) of responders
1	Hearing tests (n = 87) (Mean = 4.4, SD = 0.9)	1 (no effect)	2 (2.3%)
		2	1 (1.2%)
		3	7 (8.1%)
		4	24 (27.6%)
		5 (very effective)	53 (60.9%)
2	Completing questionnaires (n = 83) (Mean = 4.1, SD = 1)	1 (no effect)	1 (1.2%)
		2	7 (8.4%)
		3	16 (19.3%)
		4	4 (25.3%)
		5 (very effective)	38 (45. 8%)
3	Education and information about your ears as well as tinnitus/hyperacusis (n = 90) (Mean = 4.5, SD = 0.8)	1 (no effect)	1 (1.1%)
		2	1 (1.1%)
		3	6 (6. 7%)
		4	27 (30%)
		5 (very effective)	55 (61.1%)
4	Counselling (i.e. therapists listening empathically to your concerns and story) (n = 88) (Mean = 4.7, SD = 0.6)	1 (no effect)	0 (0%)
		2	0 (0%)
		3	5 (5.7%)
		4	18 (20. 5%)
		5 (very effective)	65 (73.9%)
5	Cognitive behavioural therapy (i.e. therapist working collaboratively with you to help modifying negative thoughts and feeling about tinnitus/hyperacusis) (n = 75) (Mean = 4.4, SD = 0.7)	1 (no effect)	0 (0%)
		2	0 (0%)
		3	11 (14. 7%)
		4	22 (29.3%)
		5 (very effective)	42 (56%)
6	Bedside sound generator (n = 44) (Mean =4, SD =1.4)	1 (no effect)	5 (11.4%)
		2	2 (4.6%)
		3	4 (9.1%)
		4	10 (22.7%)
		5 (very effective)	23 (52.3%)
7	Wideband noise generator (n = 59) (Mean =3.6, SD = 1.4)	1 (no effect)	7 (11.9%)
		2	4 (6. 8%)
		3	17 (28.8%)
		4	10 (17%)
		5 (very effective)	21 (35.6%)
8	Hearing aids (If you have hearing loss too) (n = 55) (Mean = 3.8, SD = 1.2)	1 (no effect)	4 (7.3%)
		2	4 (7.3%)
		3	12 (21.8%)
		4	16 (29.1%)
		5 (very effective)	19 (34.6%)
9	Overall satisfaction from the tinnitus/hyperacusis clinic (n = 88) (Mean = 4.5, SD = 0.7)	1 (no effect)	0 (0%)
		2	2 (2.3%)
		3	5 (5.7%)
		4	32 (36.4%)
		5 (very effective)	49 (55.7%)

There was no significant difference between the scores for education and counselling (*p* = 0.06) or education and CBT (*p* = 0.24). However, scores for education were significantly greater than scores for WNGs (*p* <0.001) and hearing aids (*p* <0.001). Scores for counselling were significantly higher than scores for CBT (*p* <0.001), WNGs (*p* <0.001), and hearing aids (*p* <0.001); and scores for CBT were significantly higher than scores for WNGs (*p* <0.001) and hearing aids (*p* <0.005). The scores for the bedside SGs were lower, but not significantly so, than scores for counselling (*p =* 0.007), education (*p* = 0.03) or CBT (*p* = 0.06).

A comparison of scores for responders with tinnitus only and those with hyperacusis (with or without tinnitus) showed that there were no significant differences for education (*p* = 0.32), counselling (*p* = 0.14), CBT (*p* = 0.05), bedside SGs (*p* = 0.16), WNGs (*p* = 0.29), or hearing aids (*p* = 0.26).

The mean scores given by patients for counselling (*p* = 0.92), education (*p* = 0.39), CBT (*p* = 0.9), bedside SGs (*p* = 0.22), WNGs (*p* = 0.66), and hearing aids (*p* = 0.04) did not differ significantly across audiologists, indicating that the audiologists were similar in their abilities to deliver these interventions.

As shown in Table 3, among those who rated the effectiveness of education as 4/5 or 5/5, 20% (8/39) rated bedside SGs as 3/5 or below, 45% (25/55) rated WNGs as 3/5 or below, and 35% (18/51) rated hearing aids as 3/5 or below. This indicates that many responders who did not benefit from SGs, hearing aids, or WNGs, still benefited from education. However, all except two of the responders (out of 33) who rated the effectiveness of the bedside SGs as 4/5 or 5/5, all except from one (out of 31) of those who rated the effectiveness of WNGs as 4/5 or 5/5, and all of those (*n* = 33) who rated the effectiveness of hearing aids as 4/5 or 5/5, also rated the effectiveness of education as 4/5 or 5/5. In other words, education was rated as effective regardless of the effectiveness of SGs, WNGs, or hearing aids. The outcome was similar for counselling. Among those who rated the effectiveness of counselling as 4/5 or 5/5, 25% (10/40) rated bedside SGs as 3/5 or below, 46% (25/54) rated WNGs as 3/5 or below, and 36% (18/50) rated hearing aids as 3/5 or below. All responders except three (out of 33) who rated the effectiveness of the bedside SGs as 4/5 or 5/5, all of those who rated the effectiveness of WNGs as 4/5 or 5/5 (*n* = 29), and all except one (out of 33) of those who rated the effectiveness of hearing aids as 4/5 or 5/5, also rated the effectiveness of counselling as 4/5 or 5/5.

## Discussion

### Study limitations

This was a service-evaluation survey assessing patients’ perspectives about the effectiveness of the treatments they received. This study was not designed to assess the effectiveness of the treatments, for which a randomized controlled design is required (Pocock, 1983). Also, the survey design did not allow us to assess the effects of any possible interactions between treatments. The questionnaire used was specifically designed for local service evaluation at RSCH and no data with regard to its psychometric properties are available (e.g. test-retest reliability). Therefore, our results need to be interpreted with caution.

Overall, the responders seemed to be reasonably happy with all of the interventions provided at THTSC. Although there were no significant differences in the self-report severity of the symptoms related to tinnitus or hyperacusis between the responders and non-responders, there were significant differences with regard to their age, PTA in the better ear, and the number of therapy sessions received at the time of the survey. This increases the risk of selection bias (Choi & Pak, 2005; Pannucci & Wilkins, 2010). People who are generally pleased with their treatment and the service provided may be more likely to return their questionnaires than those who are dissatisfied. It is also possible that the responses to the questionnaires were biased towards what the responders believed to be desired by the investigators (Choi & Pak, 2005). In this study, selection bias was reduced by sending the survey questionnaire to all patients seen during a three month period. However, the response rate was only about 46%. This was better than the 24% return rate in a survey conducted by other NHS audiology departments (Kelly et al, 2013), but not as high as the average response rate of 55% in the surveys conducted by primary health care services (Grol et al, 1999). The response rate might have been increased by contacting non-responders and encouraging them to return their questionnaires, but this was not possible due to resource limitations. Therefore, the outcomes of this study may not be representative of the whole sample of tinnitus and hyperacusis patients, and our findings need to be interpreted with some caution.

### Counselling, CBT, and education

Responders rated counselling, education, and CBT, as more effective than hearing aids and WNGs. This is consistent with previous reports suggesting that CBT and education have a stronger evidence base for the management of tinnitus and hyperacusis than sound therapy and hearing aids (Tunkel et al, 2014; Hesser et al, 2011). However, to the authors’ knowledge no previous study has assessed patients’ views of the effectiveness of client-centred counselling in the management of tinnitus and hyperacusis. Our study showed that client-centred counselling was rated as slightly better than CBT and as much effective as education. Although the good practice guide in the UK recommends that ‘all members of teams working with patients with tinnitus need to be competent in counselling and psychological support skills' (p.14, line 12), the document did not define exactly what was meant by counselling and psychological support. This could range from providing reassurance and information to the use of client-centred counselling. There is a discrepancy between audiologists’ perception of counselling and the client-centred counselling approach. In many audiology textbooks and research papers, counselling is described as explaining and providing technical information to the patient (English et al, 2000). However, in the context of a client-centred approach, counselling is a process that should allow the patient, not the clinician, to talk about their concerns and emotions (Rogers, 1962). The counsellor should use empathic listening skills to help the patient explore their feelings and support them in finding their own insight and solutions to the problem (Merry, 2002). Unlike the concept in audiology, counsellors commonly do not give advice, as the counselling is based on the concept that the solution to the problem lies within the patient. A clinical implication is that audiologists may need further training in the application of counselling skills to help them offer therapies for patients with tinnitus and hyperacusis. However, further research is needed to systematically assess the effectiveness of client-centred counselling (as opposed to just listening sympathetically and giving advice to the patient) in the management of tinnitus and hyperacusis.

### Bedside SGs, hearing aids, and WNGs

More than 50% of the responders rated the bedside SGs, hearing aids, and WNGs as 4/5 or 5/5. However, as sound therapy devices were always offered together with counselling and education, it is not clear whether the high satisfaction of this 50% was directly related to the effectiveness of the devices or to satisfaction with the overall therapy. Between 20% and 46% of responders who found education or counselling to be effective, rated the bedside SGs, WNGs, and hearing aids as 3/5 or below. This indicates that many patients who did not benefit from the SGs, hearing aids, or WNGs still benefited from the educational and counselling components. However, almost all of the patients who found SGs, WNGs, and hearing aids to be effective, also rated counselling or education as effective (4 or 5). This makes it difficult to determine whether the bedside SGs, hearing aids, or WNGs were effective components of the treatment package.

## Conclusions

From the patients’ perspectives, counselling was the most effective treatment in helping them to manage their tinnitus and hyperacusis, followed by education and CBT. The efficacy of CBT and education in tinnitus management has been established in previous research. However, there is a need for further research to systematically assess the effectiveness of client-centred counselling in the management of tinnitus and hyperacusis. The majority of responders who found the bedside SGs, WNGs, and hearing aids to be effective also found counselling and education to be effective. Therefore, it is not clear whether bedside SGs, WNGs, and hearing aids were important components of the treatment package.

## Appendix 1

**Table:** Service evaluation survey questionnaire

Name:Date of birth: Duration of tinnitus: …… (years) Tinnitus: Yes/No Hyperacusis: Yes/NoHearing Loss: Yes/No				
Please rank the effect of the treatments you received at the Royal Surrey County Hospital, with regard to the management of your tinnitus or hyperacusis. If you feel that you have not received a treatment, then leave that question blank.				
1. Hearing tests				
1 (no effect)	2	3	4	5 (very effective)
2. Completing questionnaires				
1 (no effect)	2	3	4	5 (very effective)
3. Education and Information about your ears as well as tinnitus/hyperacusis				
1 (no effect)	2	3	4	5 (very effective)
4. counselling (i.e. therapists listening empathically to your concerns and story)				
1 (no effect)	2	3	4	5 (very effective)
5. Cognitive behavioural therapy (i.e. therapist working collaboratively with you to help modifying negative thoughts and feeling about tinnitus/hyperacusis)				
1 (no effect)	2	3	4	5 (very effective)
6. Bedside sound generator				
1 (no effect)	2	3	4	5 (very effective)
7. White noise generator				
1 (no effect)	2	3	4	5 (very effective)
8. Hearing aids (if you have hearing loss too)				
1 (no effect)	2	3	4	5 (very effective)
9. Overall satisfaction from the tinnitus/hyperacusis clinic				
1 (no effect)	2	3	4	5 (very effective)

## Abbreviations

CBT: Cognitive behavioural therapy
HADS: Hospital anxiety and depression scale
HQ: Hyperacusis questionnaire
ISI: Insomnia severity index
NHS: National Health Service
RSCH: Royal Surrey County Hospital
SD: Standard deviation
SG: Sound generator
THI: Tinnitus handicap inventory
THTSC: Tinnitus and hyperacusis therapy specialist clinic
TRT: Tinnitus retraining therapy
ULL: Uncomfortable loudness level
VAS: Visual analogue scale
WNG: Wideband noise generator
